# A Novel Urine DNA Predictor for Noninvasive Early Diagnosis and Monitoring Minimal Residual Disease of Upper Tract Urothelial Carcinoma

**DOI:** 10.1002/cam4.70346

**Published:** 2024-10-23

**Authors:** Wei Zuo, Xuanjun Guo, Jilong Zhang, Wei Yu, Yanrui Zhang, Huanqing Cheng, Qi Tang, Xuesong Li, Zhisong He, Liqun Zhou

**Affiliations:** ^1^ Department of Urology Peking University First Hospital, Institute of Urology, Peking University, National Urological Cancer Center Beijing China; ^2^ Acornmed Biotechnology Beijing China

**Keywords:** early diagnosis, liquid biopsy, MRD monitoring, utDNA, UTUC

## Abstract

**Background:**

For early detection and postoperative monitoring of upper tract urothelial carcinoma (UTUC), the traditional detection method was limited to its invasiveness and insufficient sensitivity. We aim to use urine tumour DNA (utDNA) for detecting minimal residual disease (MRD), early diagnosis and perioperative monitoring in UTUC.

**Method:**

We previously established a utDNA multidimensional bioinformatic valuation model, named utLIFE, using low‐coverage whole‐genome sequencing and targeted deep sequencing. This prospective cohort enrolled 93 patients diagnosed with UTUC without metastasis. We collected morning urine samples on the day of surgery and the discharge day after the operation for utLIFE testing. In addition, we also enrolled 80 healthy controls to further validate the specificity of the utLIFE model in the study.

**Results:**

The utLIFE of preoperative samples could discriminate UTUC with high specificity (96.25%, 77/80), and high sensitivity (96.77%, 90/93) regardless of stage and grade. The sensitivity of utLIFE was significantly higher than urine cytology (*p* < 0.001) and fluorescence in situ hybridisation (FISH) (*p* < 0.001) (*N* = 19), especially in early‐stage and low‐grade UTUC. Postoperative utLIFE scores were significantly decreased compared with those of preoperative samples (79 vs. 36, *p* < 0.001), indicating its association with tumour burden. For special pathology types, utLIFE performed less well in sensitivity and perioperative alteration.

**Conclusion:**

In conclusion, we established a bioinformatic utDNA valuation model, utLIFE, which was validated to be a rapid and noninvasive approach with high sensitivity for early detection and MRD monitoring for UTUC.

## 1 Introduction

Upper tract urothelial carcinoma (UTUC) is uncommon and accounts for only 5%–10% of urothelial carcinoma (UC) [1]. Although various traditional methods have been used for the diagnosis of UTUC, there are still challenges. FISH and cytology were limited by their insufficient sensitivity. Approximately 20% of patients with UTUC occurs intravesical recurrence bladder urothelial carcinoma (BUC), during follow‐up assessment after radical nephroureterectomy (RNU), requiring cystoscopy evaluation for follow‐up [2]. Cystoscopy is invasive, and certain patients exhibit poor compliance with follow‐up, leading to a delay in diagnosis [3].

Minimal residual disease (MRD) refers to molecular evidence of a small amount of residual tumour cells after treatment, which can be used to assess treatment efficacy and predict the risk of recurrence. The noninvasive liquid biopsy includes urine tumour DNA (utDNA) and circulating tumour DNA (ctDNA) [4]. Multiple studies confirmed the role of ctDNA in monitoring MRD and indicating poorer prognosis [5]. Similarly, utDNA as a noninvasive detection method for monitoring UC has been one of the research's focuses currently.

Several published studies have also shown that targeted sequencing of utDNA can be a reliable alternative method for diagnosis and perioperative monitoring BUC, showing higher sensitivity and specificity than cytology [6, 7, 8]. However, utDNA as a noninvasive detection method for tumour monitoring has not been fully studied in the UTUC cohort. We still lack effective means to estimate if the patient has residual tumours. Meanwhile, previous studies also demonstrated that utDNA detection showed a strong correlation with tumour malignancy and burden [9, 10]. Our team has developed a multidimensional utLIFE model, named utLIFE, for detecting MRD and early cancer diagnosis and postoperative tumour burden monitoring in UC, demonstrating 96% specificity and 92.8% sensitivity [11]. However, utLIFE was not validated in a large cohort of UTUC patients.

In this study, we aimed to validate the utLIFE of performance in the UTUC cohort as a noninvasive early diagnosis and residual tumour monitoring after UTUC, to improve disease management.

## 2 Results

### 2.1 Patient Characterisations

A total of 104 participants patients were initially eligible for inclusion. Based on pathology diagnosis, four patients were excluded for benign tumours, two for renal tumours and five for synchronous BUC respectively. Therefore, the final number of subjects included in the study was 93 (Table 1). The average age was 68 (range, 42–91) years old. A total of 46 patients had UTUC at the pelvis, 34 at the ureter, eight at the junction of ureter and pelvis and two involving both the ureter and pelvis. The number of patients with ≥ pT2 was 45 (48.4%) and G3 was 44 (47.3%).

**TABLE 1:** Patient characteristics.

	UTUC (*N* = 93)
Sex (male, count)	55 (59.1%)
Age (median, range)	68 (42–91)
Smoke (Yes, count)	11 (11.8%)
Side (Right, count)	41 (44.1%)
Location	
Pelvis	46 (49.5%)
Ureter	34 (36.6%)
Both pelvis and ureter	2 (5.4%)
Junction of ureter and pelvis	8 (8.6%)
Pathological subtype	
UC	78 (83.9%)
UC with sarcomatoid	1 (1.9%)
UC with squamous	7 (7.5%)
UC with adenoid	2 (2.2%)
Others	5 (5.5%)
T stage	
T1	48 (51.6%)
T2	18 (19.4%)
T3	27 (29.0%)
N stage	
N0	87 (93.6%)
N1	6 (6.4%)
G grade	
G1	1 (1.1%)
G2	48 (51.6%)
G3	44 (47.3%)
SLD (median, range)	3.0 (0.9–23)
Pre utLIFE model (positive)	96.8% (90/93)
Post utLIFE model (positive)	14.0% (13/43)

**FIGURE 1:**
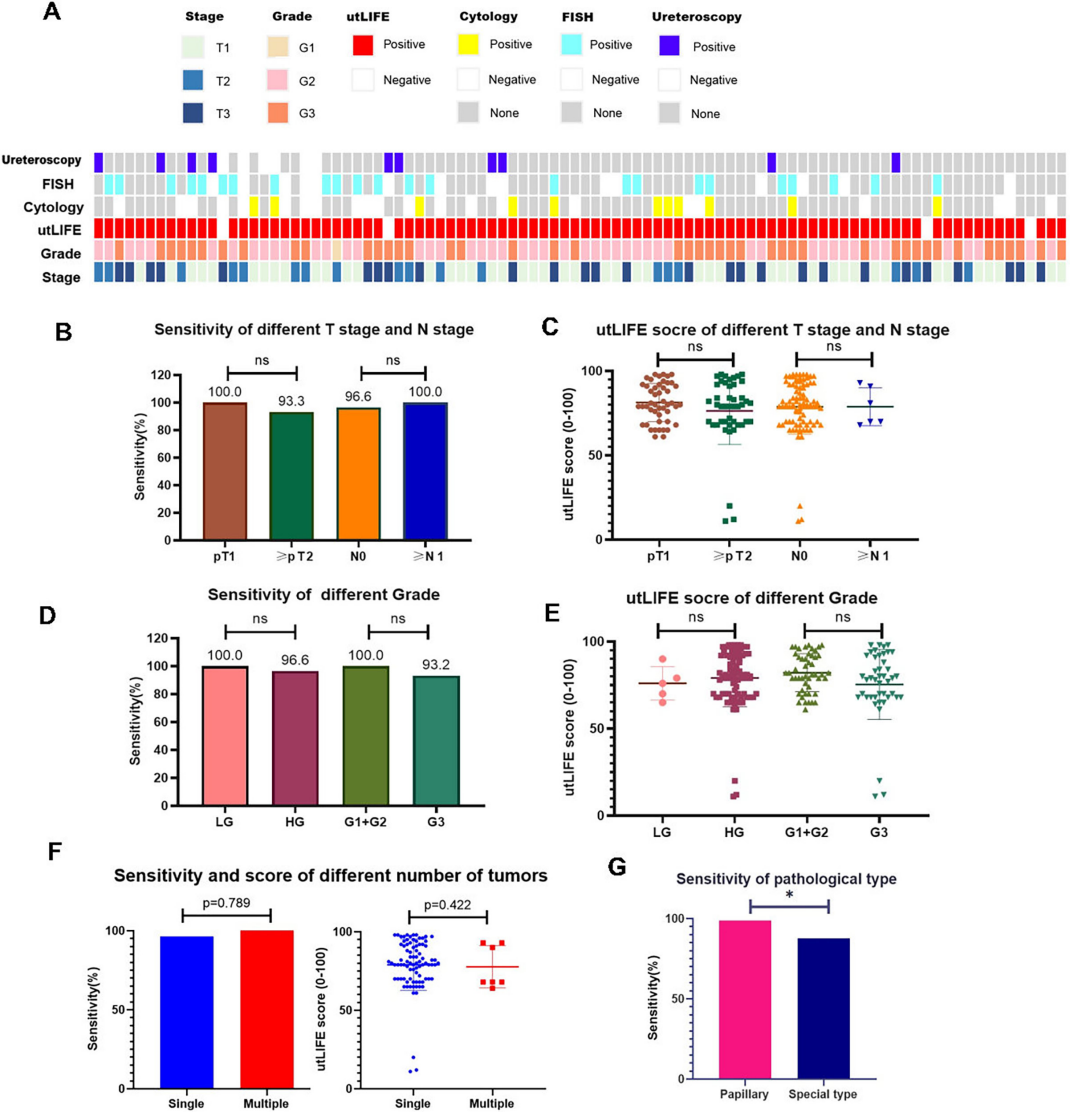
The performance of utLIFE in the diagnosis of UTUC. (A) Distribution of predicted diagnostic status using utLIFE of UTUC patients. (B–E) The sensitivity of utLIFE model to detect UTUC in different pathological T stages and grades. (F) The sensitivity of utLIFE model in different numbers of UTUC. (G) The sensitivity of utLIFE model in different pathological types.

A total of 33 and 39 patients received cytology and FISH before surgery. Sixteen patients were performed ureteroscopy and biopsy (Figure 1A).

We further enrolled 80 healthy controls and performed the utLIFE test, to validate the specificity of the utLIFE model in the UTUC cohort (supplementary table S1).

### 2.2 The Performance of the utLIFE Diagnostic Model in UTUC

The utLIFE of preoperative samples gave a diagnosis with a sensitivity of 96.8% (95%CI, 90.2%–99.2%) (90/93), with a median preoperative utLIFE score of 80 (11–98). The overall specificity is 96.3% (95%CI, 88.7%–99.0%) (77/80). The utLIFE showed an AUC of 0.957, PPV of 96.8% and NPV of 96.3%.

utLIFE reached a sensitivity of 100.0% and 93.3% (*p* = 0.109) for late‐stage (≥ pT2) and early‐stage (pT1), with a median utLIFE score of 80 (range, 61–98) and 79 (range, 11–98) respectively (*p* = 0.587). utLIFE also showed a sensitivity of 100.0% and 96.6% (*p* = 0.817) for ≥ N1 and N0, with a median utLIFE score of 75.5 (range, 68–93) and 80 (range, 11–98) respectively (*p* = 0.772) (Figure 1B,C). For histologically low‐grade (*N* = 5) and high‐grade (*N* = 97) urothelial carcinomas, utLIFE resulted in a sensitivity of 100.0% and 96.6% (*p* = 0.801), with a median score of 76 (range, 65–90) and 80 (range, 11–98) (*p* = 0.302). For ≤ G2 and G3 urothelial carcinomas, utLIFE resulted in a sensitivity of 100.0% and 93.2% (*p* = 0.102), with a median score of 81(range, 61–98) and 78.5 (range, 11–98) (*p* = 0.128) (Figure 1D,E).

The utLIFE model also showed comparable performance regardless of the number of tumours (Figure 1F). In special pathology types (squamous, glandular, sarcomatoid, clear cell, etc.), we observed that the sensitivity of utLIFE was still excellent, but it was lower than papillary UC (87.5% vs. 98.7%, *p* = 0.021) (Figure 1G). The sensitivity of utLIFE was 94.4% for small tumours (< 2cm, *N* = 18) and 97.3% for large tumours (≥ 2cm, *N* = 75) (*p* = 0.480).

Our results suggested that the utLIFE‐UC model showed excellent performance regardless of stage, grade, tumour size and the number of tumours.

### 2.3 utLIFE Showed Improved Sensitivity Compared With Urine Cytology Tests and FISH, Especially in Early‐Stage, Low‐Grade and Large‐Sized UTUC

**FIGURE 2:**
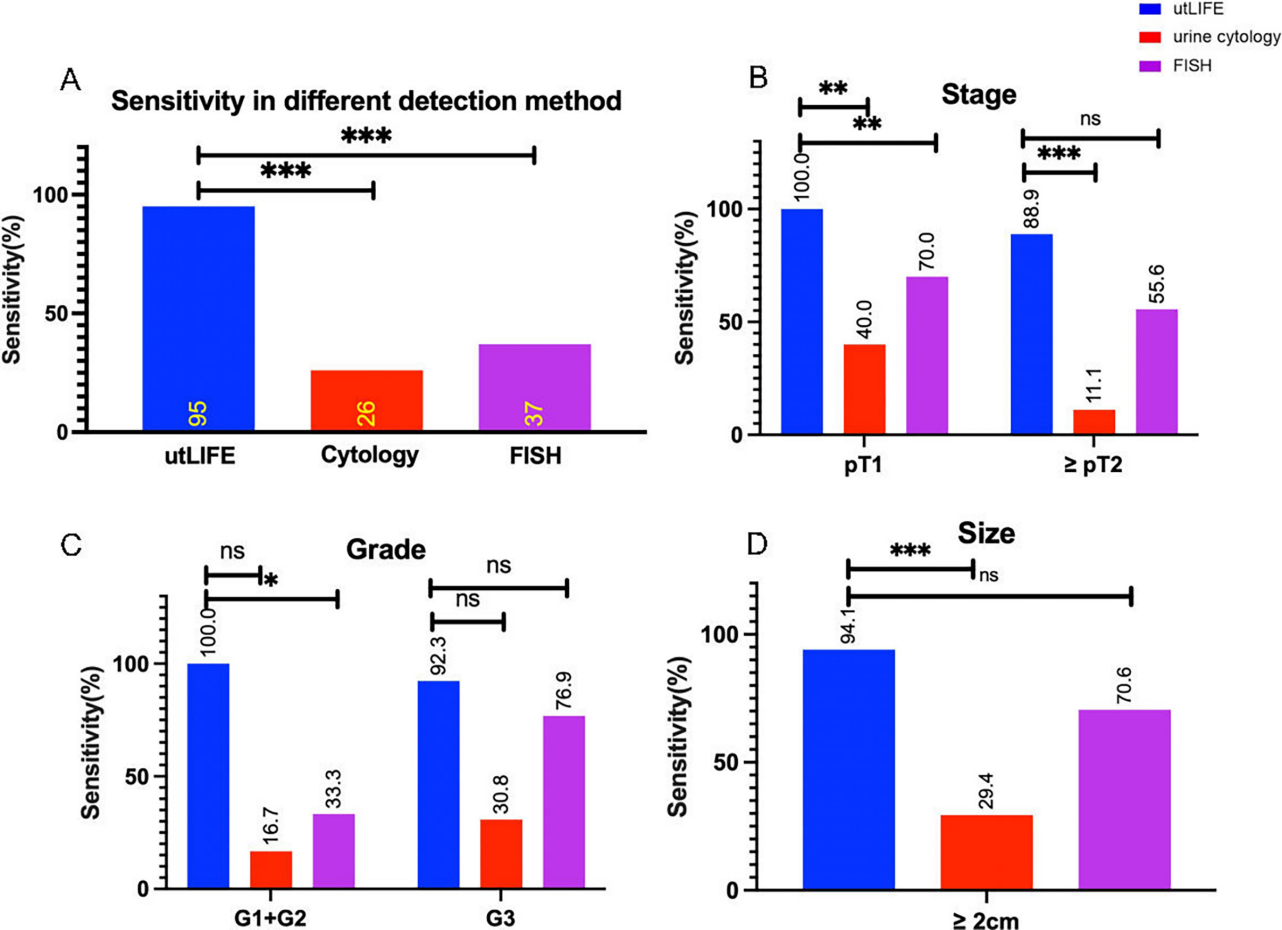
The significantly improved sensitivity of utLIFE in the diagnosis of UTUC in comparison with urine cytology and FISH. (A–D) The sensitivity of utLIFE in UTUC patients with the indicated stage (B), grade (C) and size (D) of the tumour, in comparison with urine cytology and FISH.

The landscape of clinical characteristics and the diagnostic status are shown in Figure 1A. The sensitivity of urine cytology, FISH and ureteroscopy for UC diagnosis was 33.3% (11/33), 61.5% (24/39) and 62.5% (10/16). A total of 19 patients received all utLIFE, urine cytology and FISH. The sensitivity of utLIFE (94.7%) was higher than urine cytology (26.3%, *p* < 0.001) and FISH (36.8%, *p* < 0.001). Collectively, compared with urine cytology, the utLIFE model seemed to exhibit improved sensitivity, which would be further verified in a larger prospective cohort (Figure 2A).

In both pT1 (*N* = 10) and ≥ pT2 (*N* = 9) patients, we could also observe that utLIFE showed greater sensitivity than urine cytology (T1, *p* = 0.003; ≥ T2, *p* < 0.001) However, only the sensitivity of T1 patients outperformed FISH (T1, *p* = 0.008; ≥ T2, *p* = 0.082) (Figure 2B). In both G1 + G2 (*N* = 6), utLIFE exhibited superior sensitivity than both urine cytology (*p* = 0.015) and FISH (*p* = 0.02). Otherwise, in G3 (*N* = 13) patients, utLIFE demonstrated comparable sensitivity with both urine cytology (*p* = 0.061) and FISH (*p* = 0.593) (Figure 2C).

For large tumours (≥ 2cm, *N* = 17), utLIFE showed better sensitivity than urine cytology (*p* < 0.001), but comparable sensitivity with FISH (*p* = 0.175). However, only two patients had a small UTUC tumour (< 2cm), with negative cytology, negative FISH and positive‐utLIFE (Figure 2D).

In summary, utLIFE displayed greater sensitivity than cytology and FISH in early‐stage and low‐grade UTUC. Additionally, in late‐stage, high‐grade and large‐size UTUC, the sensitivity of utLIFE was comparable to FISH.

### 2.4 utLIFE Scores Significantly Decreased After Surgery

**FIGURE 3:**
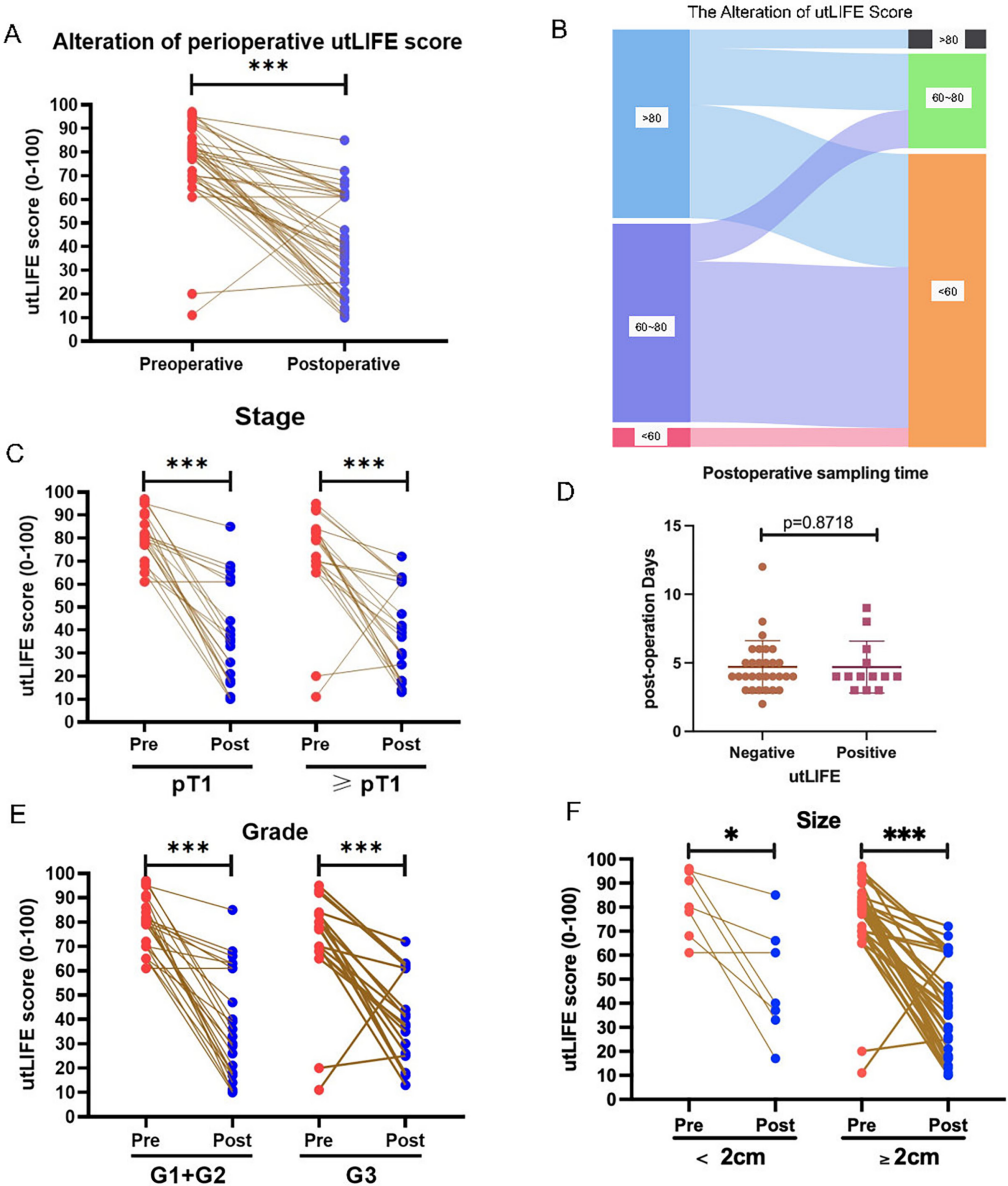
The alteration of perioperative utLIFE scores. (A, B) The alteration of utLIFE score after surgery. (D) The sampling time of utLIFE‐positive and negative patients. (C, E, F) The alteration of utLIFE score after surgery in UTUC patients with the indicated stage (C), grade (E) and size (F) of tumour.

A total of 43 patients had a postoperative utLIFE test. The sampling time of postoperative samples did not affect the test results (median = 4 days, *p* = 0.824) (Figure 3D). Postoperative utLIFE scores of MRD were significantly lower than those of preoperative samples (80 vs. 36, *p* < 0.001) (Figure 3A,B), suggesting that utLIFE may serve as an indicator of tumour burden.

Regardless of pT1 (80 vs. 35, *p* < 0.001, *N* = 21) or ≥ pT2 (79 vs. 38, *p* < 0.001, *N* = 22), a significant decrease in utLIFE scores could be both observed (Figure 3C). No matter of high‐grade (G3, 78.5 vs. 38, *p* = 0.001, *N* = 19) and low‐grade (G2, 81 vs. 31.5, *p* < 0.001, *N* = 24) patients, utLIFE reduced after the operation (Figure 3E). For large tumours (≥ 2 cm, 80.0 vs. 32.5, *p* < 0.001, *N* = 36) and small tumours (< 2 cm, 77.0 vs. 40.0, *p* = 0.028, *N* = 7), utLIFE still decreased postoperation (Figure 3F).

In special pathology types, there were no significant changes in the utLIFE score pre and postoperation (*p* = 0.15, N = 7). Nonetheless, for papillary UC, the utLIFE score reduced after surgery (79.5 vs. 36.5, *p* < 0.001, N = 36).

All 43 patients achieved surgical success and negative surgical margin. Only two patients occurred bladder recurrence at 3 months after surgery, with a postoperative utLIFE score of 18 and 21 respectively. During subsequent follow‐up (median:12.3, range 3.0–22.5 months), a total of five patients developed bladder recurrence. We found that the postoperative utLIFE score was irrelevant with the time to recurrence (*p* = 0.178, *N* = 5), and utLIFE scores are comparable (*p* = 0.672) between recurrent (*N* = 5) and nonrecurrent patients (*N* = 38).

## 3 Methods

### 3.1 Patients and Study Design

**FIGURE 4:**
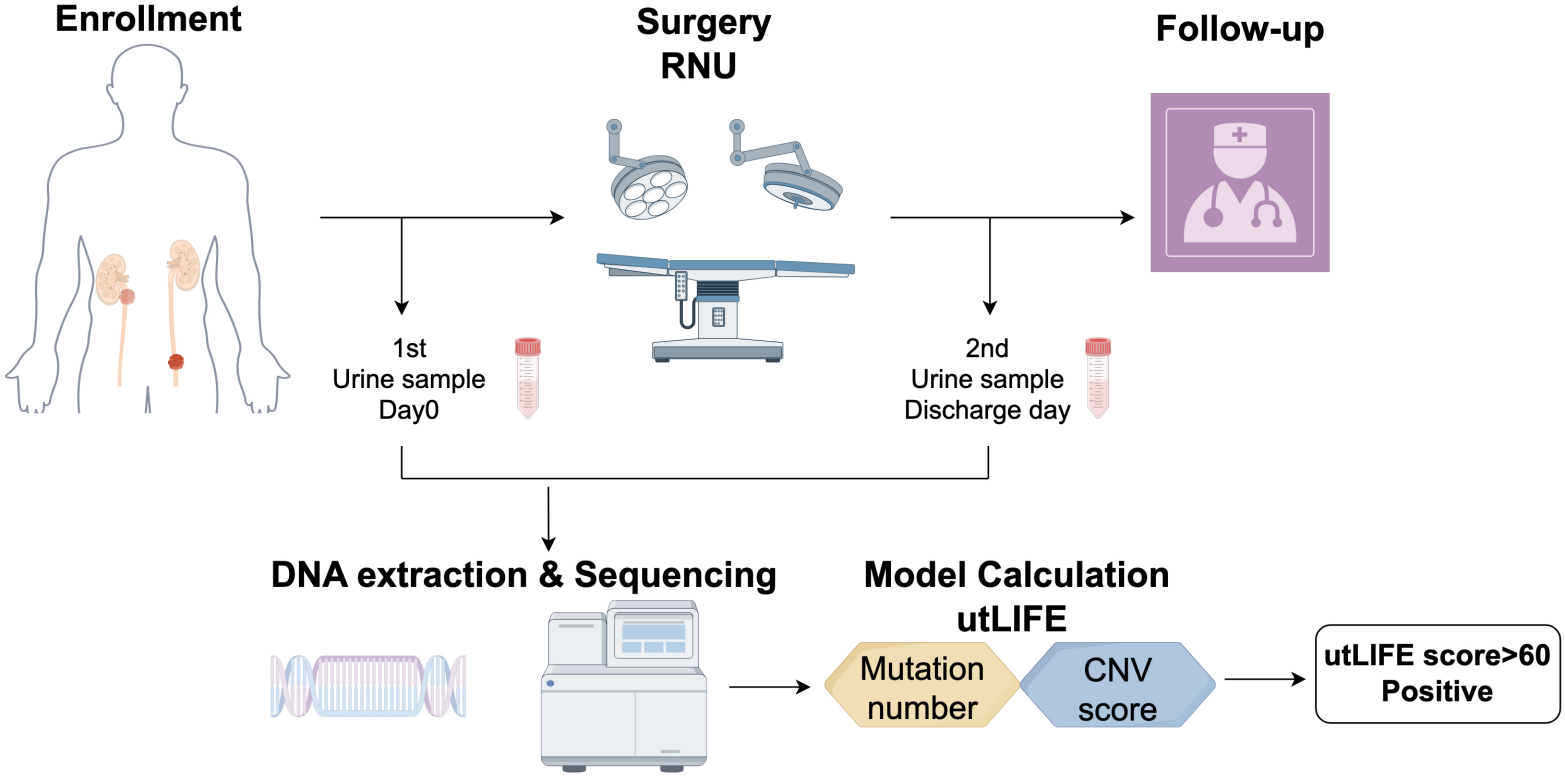
The workflow indicates study design of utLIFE.

Participants were prospectively recruited from August 2022 to January 2024 as approved by the Ethics Committee of Peking University First Hospital (NO. 2022–544). A flow diagram summarising the study design is shown in Figure 4. The inclusion criteria included patients who were diagnosed with UTUC by pathology, underwent RNU surgery, were without previous tumour disease, and were willing to attend the study by providing morning urine. Exclusions were age < 18 years, with synchronous BUC and distant metastasis.

We collected the morning preoperative urine samples on the day of surgery, and the postoperative urine samples on the discharge day. All patients underwent ultrasound inspection, CT scan or cystoscope at 3 months after surgery. We followed up with all patients until recurrence or death. The test results were not shared with surgeons and not used in management decisions. Cytology or FISH tests were decided by surgeons according to the clinical symptoms and imaging manifestations of the patients. In addition, we also enrolled 80 healthy volunteers as controls to further validate the specificity of the utLIFE model in the study, whose morning urine samples were collected for the test. We previously established a multidimensional utDNA bioinformatic valuation model, named utLIFE, using low‐coverage whole‐genome sequencing as well as targeted deep sequencing based on systematic machine learning in BUC patients. As a measure of relative importance, the proportional contributions to the algorithm score variance were calculated. The detailed procedure for building utLIFE was described in our earlier study [11]. The score of utLIFE above 60 was defined as utLIFE positive.

### 3.2 Sample Processing

Urine supernatant was collected with a urine DNA Storage Tube (CWBIO) to extract urinary cell‐free DNA (ucfDNA). The remaining urine sample was collected utilising a sterile tube with urine conditioning buffer (UCB, ZYMO) to extract exfoliated cell DNA (uexDNA). All the samples were transported at 2°C ~ 8°C conditions to the laboratory within 3 days. Quality control was evaluated by Micro Drop (BIO‐DL) and Qubit 4.0 (Thermo Fisher Scientific) instruments. A total of 1–30 ng of ucfDNA and 100 ng of uexDNA were applied to generate sequencing libraries. Sequencing was performed using Novaseq6000 platform (Illumina) in 150PE mode.

### 3.3 NGS Processing

After removing the low‐quality sequencing data, sequencing reads were aligned to the hg19 version of the reference human genome with Burrows‐Wheeler Aligner (BWA, version 0.7.12). MarkDuplicates tool in Picard was used to mark PCR duplicates. Realignment and recalibration of the BWA alignment results were evaluated by the IndelRealigner and BaseRecalibrator tool in the Genome Analysis Toolkit (GATK; version 3.8). Sentieon TNhaplotyper was utilised to identify somatic mutations.

### 3.4 Mutation Analysis

Variants with East Asian population frequency > 0.001 in the GnomAD or ExAC_EAS database were removed. The identified variants were annotated with ANNOVAR and then classified as silent or nonsilent types. The silent types contained exonic synonymous variants, intronic variants, 3'UTR variants, 5'UTR variants and promoter variants (except the TERT promoter). The nonsilent types contained exonic nonsynonymous variants, stop codon variants, splicing variants, frameshift insertion variants, nonframeshift insertion variants, frameshift deletion variants and nonframeshift deletion variants. All the silent variants were filtered out. Nonsilent variants with mutant allele frequency (MAF) < 0.01, allele base depth < 3 and reference base depth < 10 were excluded. Finally, we performed target deep sequencing of the 155 genes using ucfDNA, which were selected from the previous study, including the two most commonly mutated genes of *TERT* and *TP53* and other genes with a high frequency of variants, such as *ERBB2*, *ERCC2* and *FGFR3* [11].

### 3.5 Copy Number Variation (CNV) Analysis

Large CNVs were analysed based on the shallow whole‐genome sequencing (1 × WGS) data of uexDNA. The coverage was measured for every 200 k bin for all samples, which was further corrected by GC context and self‐standardisation. The normal samples served as control to remove centromeres, telomeres and repeat regions with no genetic information or considerable noise. All the samples were analysed according to the following algorithm after standardisation:

$$N=\sum \text{segment length}\left(\left|\log_{2}\text{ratio}\right|,>\text{Cutoff}\_1\right)$$

If the absolute value of the segment is greater than Cutoff_1, then the segment is considered to have CNV events [11].

### 3.6 Calculating utLIFE Score

The utLIFE model incorporated a comprehensive feature matrix encompassing both genetic alterations and copy number variations (CNVs). This matrix serves as the input for our classification model, which is fundamentally an SVM (support vector machine) model. The raw output score of the utLIFE model spans a continuum from 0 to 1, reflecting the probability of urothelial carcinoma. The established threshold for the model is determined to be 0.6 identified according to the maximum Youden Index value, a particular datum that remained unreported in our previous study [11]. To enhance comprehensibility for both medical professionals and patients, we have scaled this score by multiplying it by 100, thereby transforming the range to a more intuitive scale of 0 to 100, and the threshold changed to 60. If the adjusted utLIFE score is equal to or below the predetermined threshold (score: 60), the individual is classified as ‘negative’, indicating a decreased likelihood of having urothelial carcinoma. Conversely, scores exceeding 60 are interpreted as ‘positive’, suggesting a diagnosis of urothelial carcinoma. This binary categorisation aids in the clinical decision process, offering a clear and actionable interpretation of the model's predictive output.

### 3.7 Statistical Analyses and Data Visualisation

All statistical analyses were performed using SPSS26.0. Data are reported as means and SDs, medians and interquartile ranges, and HRs or ORs with 95% confidence intervals (CIs), as appropriate. The McNemar test was used to compare the sensitivity of different methods. Missing data were removed from the analyses. All analyses were performed with the use of R software, version 3.4.3 (R Foundation for Statistical Computing).

## 4 Discussion

Our study demonstrates that utLIFE provides a promising approach for accurate noninvasive UC screening, surpassing the current standard procedure in clinical practice. Thus, utLIFE might be an alternative to ureteroscopy for noninvasive diagnosis. Furthermore, the utLIFE score may reflect the tumour burden in real time, showing the possibility of recurrence monitoring after surgery. Taken together, utLIFE demonstrated practical clinical utility in early detection and MRD monitoring.

Nowadays, liquid biopsy has been widely used in UC due to its high sensitivity and noninvasive approach. The liquid biopsy includes CTC, ctDNA, circulating tumour RNA (ctRNA), utDNA, extracellular vesicles (EV), proteomics and metabolomics [12]. As a predominant proportion, utDNA has gained great attention for detecting UC, which is rich in sources, easy to obtain and relatively less contaminated. Besides, the consistency between utDNA and tumour DNA is higher than ctDNA [6, 13]. However, there is still a lack of common mutations in all UC patients. In addition, as a hallmark of human cancer, chromosomal alterations have proven their potential utility in the detection of UC, but they yet rarely applied in liquid‐biopsy of utDNA [14, 15, 16]. To combine the detection ability of multidimensional features, we develop a novel noninvasive urine test called utLIFE, which consists of genetic alterations and large copy number variants (CNVs) with a customised bioinformatics workflow [11]. utLIFE diagnostic model is a cost‐effective, rapid, high‐throughput, noninvasive and promising approach for early diagnosis and residual disease detection in UC. Of note, our MRD detection does not require prior sequencing of tumour tissue.

In the past few years, it has been demonstrated that genomic, transcriptomic, epigenomic and macrogenomic studies for utDNA have shown high diagnostic efficacy and recurrence monitoring capabilities in UC [7, 8, 9, 10]. However, utDNA as a noninvasive detection method for tumour monitoring has not been fully studied in the UTUC cohort. Recently, a utDNA tool, EpiCheck, achieved 83% sensitivity and 79% specificity, significantly outperforming cytology in UTUC [17]. In our study, the utLIFE model possessed high accuracy and strong clinical utility in the detection of UTUC, with a sensitivity of 96.77%, which is about twofold higher than the urine cytology and the FISH test. Diagnosing early‐stage and minimal tumours of UTUC was highly challenging, and often overlooked by cytology and FISH. Notably, utLIFE showed significant superiority compared to FISH, offering a promising solution for early‐stage and low‐grade tumours. For our patient with a small tumour, the sensitivity of utLIFE was also relatively high and utLIFE probably offers an effective reference for this condition, even taking the small sample size into account.

Liquid biopsy based on ctDNA assessment of MRD and prediction of recurrence risk has been widely explored and applied in pancreas, breast, lung cancers and muscle‐invasive UC for predicting metastatic recurrence, prognosis and even therapeutic efficacy [5, 18, 19, 20], which suggests the value for the assessment of MRD with solid tumours. However, studies detecting MRD based on utDNA are still lacking. In addition, utDNA methylation assessment tools have also shown that utDNA shares a strong correlation with tumour malignancy and burden [7, 9]. Our study demonstrated that postoperative utLIFE MRD scores decreased after surgery, regardless of stages and grades, indicating that utLIFE was associated with tumour burden in real‐time. The Imvigor010 trial found that among ctDNA‐positive patients, both disease‐free survival (DFS) and overall survival (OS) were significantly improved in the atezolizumab‐treated group compared with the observation group [5, 21]. Because the Imvigor010 trial included UTUC less than 10%, it is suggested that ctDNA might also be a biomarker to predict the prognosis of postoperative adjuvant therapy in UTUC [21]. Moreover, it is reported that there are differences in DNA alterations between BUC and UTUC [22, 23]. Therefore, it is necessary to verify whether the same utLIFE model is accurate for UTUC in early diagnosis, even recurrence monitoring.

There are several limitations in this study. First, the sample size is insufficient, and further increase in patients with preoperative cytology and FISH needed to improve. Second, the utLIFE model cannot differentiate between UTUC and BUC and the estimation results of the utLIFE may be disturbed when synchronous UTUC‐BUC. Besides, the sensitivity in patients with special pathology types was slightly inferior compared to papillary UC, which needs a further improved model. The healthy controls enrolled in this study were healthy volunteers. This may lead to an overestimate of specificity in the study. Additionally, the threshold of score 60 was determined using the data in the current study. As there is no independent test set, there is a risk of overfitting.

Therefore, further construction of independent detection models for UTUC and BUC should be explored based on the sequencing results and their specificity should be verified. In addition, follow‐up treatment and prognosis will be tracked for subsequent studies.

## 5 Conclusion

Our study showed that uLIFE, as a noninvasive urinary DNA bioinformatics assessment model, possessed high accuracy accompanied high specificity, and strong clinical utility in the early diagnosis of UTUC, which exhibited improved sensitivity compared with cytology and FISH. In addition, the results of postoperative utLIFE showed a promising potential for perioperative monitoring and postoperative adjuvant therapy decisions.

## Author Contributions

**Wei Zuo:** formal analysis (equal), visualization (equal), writing – original draft (equal). **Xuanjun Guo:** writing – review and editing (equal). **Jilong Zhang:** data curation (equal), writing – original draft (equal). **Wei Yu:** data curation (equal). **Yanrui Zhang:** methodology (equal). **Huanqing Cheng:** methodology (equal). **Qi Tang:** funding acquisition (equal), project administration (equal), resources (equal), writing – review and editing (equal). **Xuesong Li:** conceptualization (equal). **Zhisong He:** conceptualization (equal). **Liqun Zhou:** conceptualization (equal).

## Ethics Statement

The authors are accountable for all aspects of the work in ensuring that questions related to the accuracy or integrity of any part of the work are appropriately investigated and resolved. The trial was conducted by the Declaration of Helsinki (as revised in 2013). This study was approved by the institutional review board of Peking University First Hospital (NO: 2022–544).

## Consent

Informed consent was obtained from all subjects involved in the study.

## Conflicts of Interest

The authors declare no conflicts of interest.

## Supporting information


**Table S1.** Baseline characteristics of healthy controls.

## Data Availability

The datasets used and analysed during the current study are available from the corresponding author upon reasonable request.
