# Supplementary material for: A Novel Urine DNA Predictor for Noninvasive Early Diagnosis and Monitoring Minimal Residual Disease of Upper Tract Urothelial Carcinoma
Source: Cancer Med. 2024 Oct 23;13(20):e70346. doi: 10.1002/cam4.70346 (PMC11497171; doi:10.1002/cam4.70346)
Supplement: Supplementary file 1 — Table S1. Baseline characteristics of healthy controls. [file CAM4-13-e70346-s001.docx]

Supplementary Table 1. Baseline characteristics of healthy controls.

|  | Healthy control  (N=80) |
| --- | --- |
| Sex (male, count) | 48（60.0%） |
| Age (median, range) | 37（24-83） |
| Pre utLIFE model (positive, %) | 2.5%（2/80） |
| Pre utLIFE score | 27 (10-90) |
